# Anti-cancer management of head and neck cancers and oral microbiome—what can we clinically obtain?

**DOI:** 10.3389/fcimb.2024.1329057

**Published:** 2024-02-28

**Authors:** Jakub Makarewicz, Karolina Kaźmierczak-Siedlecka, Bartosz Kamil Sobocki, Iwona T. Dobrucki, Leszek Kalinowski, Ewa Stachowska

**Affiliations:** ^1^ Scientific Circle of Studies Regarding Personalized Medicine Associated with Department of Medical Laboratory Diagnostics, Medical University of Gdansk, Gdansk, Poland; ^2^ Department of Medical Laboratory Diagnostics—Fahrenheit Biobank BBMRI.pl, Medical University of Gdansk, Gdansk, Poland; ^3^ Department of Oncology and Radiotherapy, Medical University of Gdansk, Gdansk, Poland; ^4^ Department of Bioengineering, University of Illinois at Urbana—Champaign, Urbana, IL, United States; ^5^ Beckman Institute for Advanced Science and Technology, University of Illinois at Urbana—Champaign, Urbana, IL, United States; ^6^ Department of Biomedical and Translational Sciences, Carle Illinois College of Medicine, University of Illinois at Urbana—Champaign, Urbana, IL, United States; ^7^ BioTechMed Centre, Department of Mechanics of Materials and Structures, Gdansk University of Technology, Gdansk, Poland; ^8^ Department of Human Nutrition and Metabolomics, Pomeranian Medical University in Szczecin, Szczecin, Poland

**Keywords:** head and neck cancers, oral mucositis, radiotherapy, brachytherapy, chemotherapy, microbiome

## Abstract

Head and neck squamous cell carcinoma (HNSCC) exhibits significant genetic heterogeneity and primarily concerns the oral cavity and oropharynx. These cancers occur more frequently in men with a 5-year survival rate below 50%. Major risk factors include human papilloma virus (HPV) (notably type 16), Epstein–Barr virus, tobacco, alcohol, and poor oral hygiene with approximately 4.5% of global cancers linked to HPV. Notably, differences in the microbiome between healthy individuals and patients with head and neck cancers (HNCs) have been identified. Recent studies highlight the significance of certain oral microbes in risk assessment and the potential of the microbiome as a biomarker for HNCs. Additionally, role of the microbiome in metastasis has been acknowledged. Treatment for HNCs includes local methods, such as surgery and radiotherapy, and systemic approaches, such as immunotherapy. Numerous side effects accompany these treatments. Emerging research suggests the beneficial role of preoperative immunonutrition and probiotics in patient outcomes, emphasizing the influence of the microbiome on treatment efficacy. This review explores the reciprocal effects of HNC treatment and the gut microbiome using radiotherapy, brachytherapy, surgery, immunotherapy, and chemotherapy.

## 1 Introduction

Head and neck squamous cell carcinoma (HNSCC), which present a high rate of genetic heterogeneity, is a group of neoplasms of the oral cavity, oropharynx, larynx, hypopharynx, and nasopharynx (Hübbers and Akgül, 2015; Alsahafi et al., 2019; Horton et al., 2019). Cancers of the oral cavity and oropharynx are the main types of HNSCCs (Chattopadhyay et al., 2019). According to statistics data, these types of cancers occur more frequently in men than in women (5.8 *vs*. 2.3/100,000, respectively) (Dorobisz et al., 2023). It is estimated that the 5-year survival rate is less than 50% (Dorobisz et al., 2023). Human papilloma virus (HPV) (especially type 16), Epstein–Barr virus, tobacco, usage of alcohol, and poor oral hygiene are the main risk factors of head and neck cancer (HNC) (Hübbers and Akgül, 2015; Rettig and D’Souza, 2015; Cohen et al., 2018; Kaidar-Person et al., 2018; Dorobisz et al., 2023). It is estimated that even 4.5% of cancers worldwide are associated with HPV infection (Roman and Aragones, 2021). The difference of the overall microbiome (not only viral infections) profile between healthy people and patients with HNCs was detected (Dorobisz et al., 2023). In the study of Hayes et al., it was shown that abundance of oral microbes, such as *Corynebacterium* and *Kingella*, is related to a lower risk of HNSCC (Hayes et al., 2018). The *Corynebacterium* sp. is known as a commensal microbe, and its decreased amount was reported in patients with dental caries as well as periodontitis (Treerat et al., 2020). Benjamin et al. reported that oral microbiome of patients with HNSCC is characterized by abundance of the Lachnospiraceae and Eiknella families (Benjamin et al., 2023). The results from a recently published study revealed that the salivary microbiome of patients with oral cancer has a higher amount of potential pathogens in comparison to that of healthy subjects (Mäkinen et al., 2023). Some of the microbes can be established as a biomarker allowing to prognose the development of HNCs (Dorobisz et al., 2023). It has been also recognized that the microbiome affects the metastasis process (Miranda-Galvis et al., 2021). Recently in 2024, Chen et al. found that outer-membrane vesicles secreted by *Fusobacterium nucleatum* are able to induce oral cancer metastasis (Chen et al., 2024). They activate intracellular autophagy pathways. Notably, the blockage of autophagic flux using the autophagy inhibitor chloroquine decreased the migration of cancer cells, which was previously stimulated by these outer-membrane vesicles (Chen et al., 2024).

The risk of oral squamous cell carcinoma (OSCC) is higher in patients with premalignant lesions. Khan et al. analyzed the molecular mechanisms, which are the basis of progressions of premalignant lesions to OSCC and can be helpful in the detection of pathological changes in the early stage (Khan et al., 2023). In this study, datasets of gene expression and microbial profiles of oral tissues from patients presenting premalignant lesions were investigated. These profiles were compared with profiles of OSCC and normal oral mucosa. In that context, it was noted that there are similarities between OSCC and premalignant lesions (Khan et al., 2023). The potency of oral microbiome signatures as a noninvasive biomarker has been recently also demonstrated in the study of Yu et al. on patients with laryngeal squamous cell carcinoma (Yu et al., 2023).

The management of HNCs can basically be divided into two types, i.e., local and systemic (such as immunotherapy and chemotherapy). HNCs can be treated locally by radiotherapy, brachytherapy, and surgery. Nevertheless, the main treatment methods are mostly surgical eradication and radiotherapy (Alsahafi et al., 2019; Bye et al., 2020). There are many side effects of anti-cancer management, such as oral mucositis, dry mouth, oral candidiasis, dysphagia, and loss of taste (Nuchit et al., 2020; Alfouzan, 2021; Loewen et al., 2021). Recently, it was shown that preoperative immunonutrition significantly decreased overall complications (*p* = 0.034) and length of hospital stay (*p* < 0.001) in HNCs patients (Mueller et al., 2019). In another randomized, double-blind, and placebo-controlled study, Jiang et al. reported that probiotics by modulation of the gut microbiome reduced the severity of oral mucositis, which is caused by chemoradiotherapy in nasopharyngeal carcinoma (Jiang et al., 2019). Currently, the influence of microbiome and its related aspects on anti-cancer treatment response is increasingly considered (not only in the context of immunotherapy), and it may support new clinical perspectives. Therefore, in the present review, we discussed the bidirectional impact of complex anti-cancer treatment of HNCs on the gut microbiome as well as the influence of the microbiome on the efficiency of HNC management.

## 2 Radiotherapy

Radiation therapy (RT) is used to treat most types of cancers, often in combination with other methods (Siegel et al., 2021). Notably, radiotherapy is considered as a curative-intent treatment of HNCs (Alterio et al., 2019). Despite the fact that the abovementioned therapy is often crucial in the treatment of HNCs, it is associated with many complications occurring especially in the oral cavity and pharynx. These side effects include dry mouth as a consequence of reduced saliva production, mucositis, oral candidiasis, osteoradionecrosis of the jaw, loss of taste, caries, and periodontal diseases. It is recommended to provide an appropriate dental care and treatment (such as elimination of oral infections and extractions of teeth with poor prognosis) before the introduction of radiotherapy to avoid, among others, osteoradionecrosis (Abed et al., 2020). The definition of osteoradionecrosis is not completely provided, but four of its stages are listed based on the Lyons and Bernnan classification (stage 1: <2.5-cm length and asymptomatic exposed bone; stage 2: >2.5-cm length and asymptomatic exposed bone with pathological fracture and/or inferior alveolar nerve canal involvement; stage 3: >2.5-cm length and symptomatic exposed bone, however, with no other features despite medical treatment; stage 4: >2.5-cm length and symptomatic exposed bone with pathological fracture and/or inferior alveolar nerve canal and orocutaneous fistula) (Abed, 2023). Despite the fact that the incidence of osteoradionecrosis decreased during the last years, it is still an aggressive late complication (Kubota et al., 2021). Li et al. reported that alterations in the oral microbiota may affect osteoradionecrosis (Li et al., 2023). This study included 30 patients with HNCs who were treated with high-dose radiotherapy. Oral swabs were taken from osteoradionecrosis lesions and contralateral normal tissues, and the next microbiome was analyzed using 16S rRNA sequencing. The abundance of some microbes was detected in case of osteoradionecrosis, thus suggesting the link between it and the microbiome (Li et al., 2023). Dental care is also important in the post-radiation period including regular radiographs, relief of dry mouth, usage of high-fluoride toothpaste, and oral rehabilitation (Abed, 2023). Reduction of saliva contributes to the development of periodontitis due to environmental alterations regarding also microbiome changes (Sroussi et al., 2017). In the study of Arrifin et al., it was observed that flow rates of both stimulated/unstimulated saliva were reduced after radiotherapy (Arrifin et al., 2018). Moreover, the pH of saliva and buffering capacity were decreased in that case. It should be emphasized that a reduced rate of flow of stimulated/unstimulated saliva was correlated with an increased number of *Candida albicans* (Arrifin et al., 2018). Similarly, in another study, it was noted that xerostomia is related to yeast populations (Gaetti-Jardim et al., 2018). Additionally, symptoms, such as mucositis, dermatitis, candidiasis, and dry mouth, frequently occur during radiation (Gaetti-Jardim et al., 2018). Interestingly, it was also revealed that non-albicans *Candida* species were commonly found in cases of xerostomia in patients with HNCs (Tarapan et al., 2019). Recently, the link between changes in the oral microbiome associated with radiotherapy and caries was investigated (Mougeot et al., 2019). The analysis was conducted regarding baseline, and at 6 (T6) as well as 18 (T18) months post-radiation therapy. There were two analyzed groups according to the DMFS score as follows: (1) patients with tooth decay increase—DMFS (+) and (2) with no increase—DMFS (−). The alterations in beta diversity was noted at both T6 and T18. Notably, the relative abundance of *Streptococcus mutans* (which is known as a major agent of dental caries) was increased at T6 in both groups, whereas in the DMFS (+) group, the relative abundance of *Abiotrophia defective* (with potential protective role) was reduced (Mougeot et al., 2019). Nevertheless, the association presented in this study requires further investigation.

**Figure 1:**
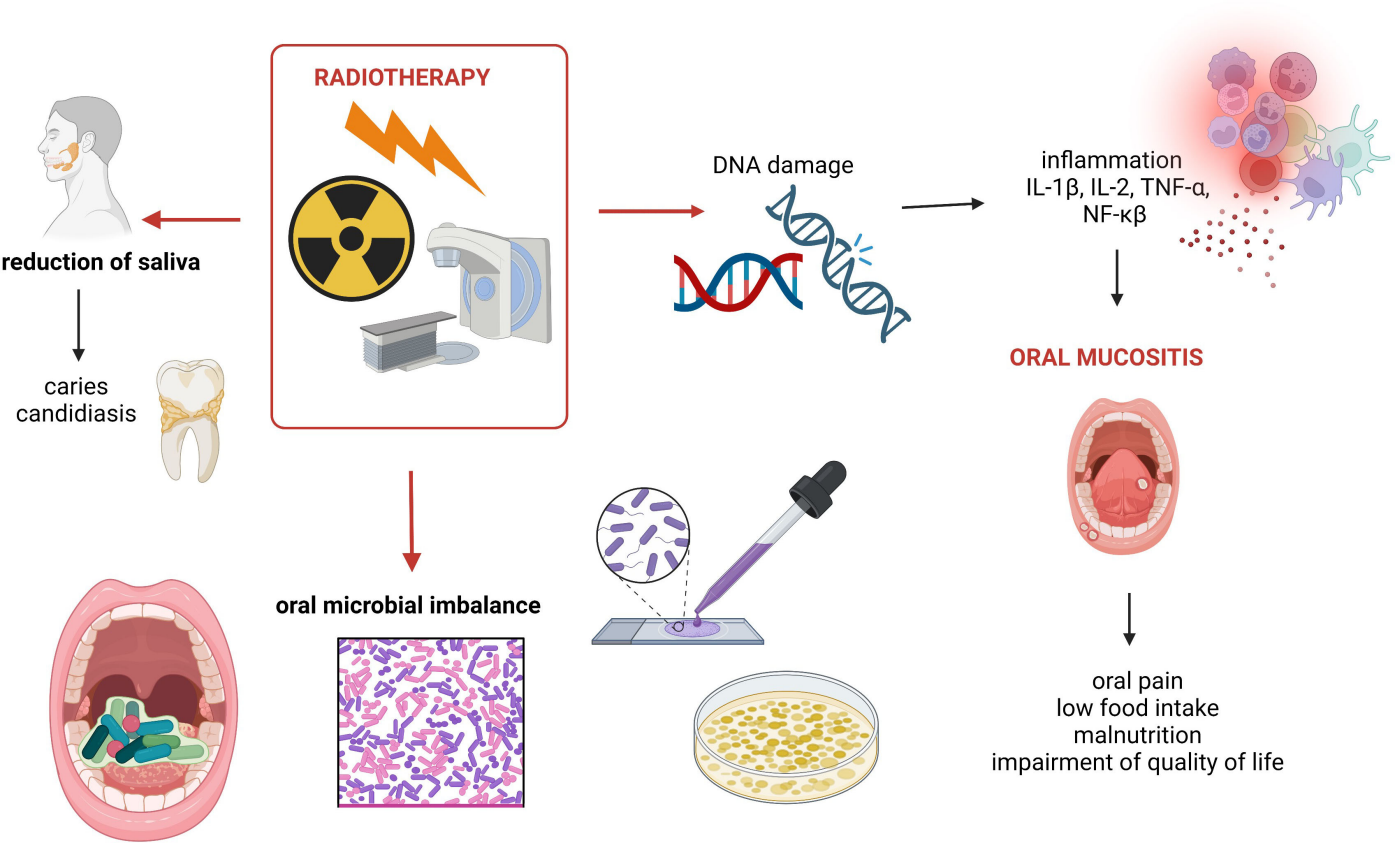
Summary of oral cavity-related side effects of radiotherapy used to treat HNCs. Own elaboration based on literature (Sroussi et al., 2017; Abed et al., 2020; Zagury-Orly et al., 2021). This figure was created using Biorender.com.

There are two mechanisms involving inflammation associated with radiation-induced microbiome changes as follows: [1] it directly causes both tissue oxidation and inflammation, consequently altering the local microenvironment and promotes microbial imbalance; [2] radiotherapy causes toxic damage to the epithelium, ulcerations, and translocation of microbes (Zagury-Orly et al., 2021). For instance, stage 4 (observed as ulcerations) is strongly associated with invasion of bacteria into submucosal and vascular compartments (Reis Ferreira et al., 2022). Overall, radiation directly and indirectly causes DNA damage leading to epithelial cell death, ulceration, and inflammation (Wakamori et al., 2022). In that process, the release of pro-inflammatory mediators, such as IL-1β, TNF-α, and NF-κβ, is significant (Lee and Galloway, 2022). Notably, oral dysbiosis is linked to both local and systemic multiple disease/condition occurrence. It can be involved in the development of not only periodontitis, dental caries, and HNC but also in endocarditis, atherosclerosis, and many others (Radaic and Kapila, 2021) (**Figure 1**).

As was mentioned above, oral mucositis is one of the side effects of radiotherapy. Oral epithelium breakdown and translocation of pathogens are observed (Al-Qadami et al., 2022). It is estimated that radiation-induced oral mucositis occurs in up to 80% of patients with HNCs (Maria et al., 2017). Oral mucositis is associated with not only oral pain but also with many complications regarding impairment of quality of life and low food intake, which consequently leads to the development of malnutrition. Alterations in food intake are especially observed in the case of oral mucositis from grade 2 and above. According to the Common Terminology Criteria for Adverse Events Version 5.0, in grade 2 oral mucositis, a modified diet is recommended, whereas in grade 3 oral mucositis, severe pain occurs, and it significantly affects oral intake (Lee and Galloway, 2022). Recently, in 2024, Hes et al. investigated the link between the gut microbiome and the side effects of chemoradiotherapy HNSCC (n = 52) (Hes et al., 2024). It was noted that all participants had mucositis induced by chemoradiotherapy, whereas 42% presented severe events—grade ≥3. The difference in microbial beta diversity was noted between patients with severe mucositis and patients with grades 1 to 2 mucositis. Moreover, the shorter overall survival was observed in patients with severe mucositis (HR = 3.3, 95% CI 1.0–10.6; *p* = 0.02) (Hes et al., 2024). The personalized treatment of oral mucositis can be based on the potency of the microbiome (Zhang et al., 2024). Oral mucositis can be prevented using probiotics (Xia et al., 2021). In a mouse model study, it was shown that oral administration of the probiotic *Streptococcus salivarius* K12 changed beneficially the oral microbiome and alleviated oral mucositis induced by radiotherapy (Wang et al., 2021). This probiotic strain was previously analyzed and recommended in the case of halitosis as well as oral candidiasis. Other studies revealed that the severity of oral mucositis may be reduced by maintaining an appropriate oral hygiene and supplementations (Lalla et al., 2019). It is noteworthy that recently, in systematic review, it was shown that benzydamine hydrochloride mouth rinse cannot mitigate radiation-induced oral mucositis, whereas oral glutamine is promising in that context (Davy and Heathcote, 2021). Similarly, in another systematic review and meta-analysis (which meets the PRISMA guidelines, n = 922), it was reported that oral glutamine supplementation may both prevent and ameliorate radiation-induced oral mucositis in patients with HNCs (Alsubaie et al., 2021). The oral microbiome prior to the introduction of radiotherapy affected the severity of oral mucositis, which has been shown in a pilot study on 20 patients with HNCs (Al-Qadami et al., 2023). In a Chinese study (Zhu et al., 2017), the effect of oral microbiota on prediction of oral mucositis in patients with nasopharyngeal carcinoma was investigated (n = 41). The oral microbiota was analyzed using 16S rRNA gene sequencing (Zhu et al., 2017). It was noted that changes in the oral microbial community correlated with the progression of radiotherapy-induced mucositis. Therefore, it can be useful for the early prediction and prevention of radiation-induced mucositis (Zhu et al., 2017). In another study, the association between oral microbiome and oral mucositis in patients with HNCs has been investigated (Vesty et al., 2020). In that study, saliva and oral swabs were taken. Considering the presence of fungi, *Candida* was widely detected in buccal mucosa swabs, despite the status of oral mucositis. It was also noted that several specific microbes (*Bacteroidales* G2, *Capnocytophaga*, *Eikenella*, *Mycoplasma*, *Sneathia*, *Porphyromonas*, *Tannerella*) were positively correlated with ≥grade 2 oral mucositis. The authors reported that patients may be more susceptible of developing oral mucositis in the case of abundance of some of the abovementioned microbes on the buccal mucosa. It is noteworthy that these bacteria—*Porphyromonas*, *Tannerella*, and *Fusobacterium*—are related to the development of periodontitis (Gasmi Benahmed et al., 2022). *Porphyromonas gingivalis* belongs to the red complex, and it is known as a major periodontopathic microbe characterized by multiple virulence factors (Chigasaki et al., 2021). The oral microbiome is altered in patients who underwent radiotherapy. A high abundance of *Streptococcus* sp. was reported in a case of oral cancer prior to radiotherapy, whereas *Klebsiella* and *Pediococcus* species as well as *C. albicans* were detected in the post-radiotherapy period (Anjali et al., 2020). In another prospective cohort study, it was observed that the number of commensal Gram-negative bacteria was reduced after exposure to radiation in the head and neck region (Mojdami et al., 2022). Schuurhuis et al. investigated the changes in microbe composition depending on the methods of treatment (total patients n = 82; n = 29 surgical treatment, n = 26 intensive modulated radiation therapy, n = 27 intensive modulated radiation therapy in combination with chemotherapy) (Schuurhuis et al., 2016). First, the changes in the oral microbiota were different considering the types of treatment. Second, the tendency of the opportunistic pathogens to increase was observed after intensive modulated radiation therapy (both with or without chemotherapy); however, it was detected in the case of surgery (Schuurhuis et al., 2016). Notably, not only oral microbiome is changed in association with radiotherapy but also microbiome of the other anatomical structures. The composition of microbiota in post-radiation sinusitis was analyzed in the study of Stoddard et al. (2019). It was shown that after radiotherapy, *Staphylococcus aureus* was the most commonly found organism followed by *Pseudomonas aeruginosa*.

Besides radiotherapy, interstitial brachytherapy is another type of local tumor treatment used in some cases of HNCs. For instance, it can be introduced with success in patients with lip cancer (Merfeld et al., 2023). Currently, in *ClinicalTrials.gov* system, 11 trials are registered, which analyze the usage of brachytherapy in HNCs, and none of them were regarding microbiome aspects. Nevertheless, the changes in the oral microbiome can be suspected because brachytherapy is given locally in a low or high dose. It could be beneficial to investigate brachytherapy in that context, which has not been studied yet.

## 3 Surgery

Surgical eradication plays a pivotal role in the management of HNCs. In the early stages of oral squamous cell carcinoma, surgery and radiation therapy were a major combination, often linked with chemotherapy based on cisplatin (Le and Hanna, 2018; Sami et al., 2020). However, similarly, as in the case of other methods of anti-cancer treatment, it causes complications. The effect of surgical site infections (SSIs) on oral microbiome has been recently investigated in the study of Zenga et al. (2022). It was noted that bacteria causing SSIs were often detected in the pre-operative oral cavity (Zenga et al., 2022). SSI-related aspects were also analyzed in the study of Durand et al. on 484 patients with HNCs treated with free flap surgery (Durand et al., 2015). SSIs were assessed ≤30 days in the post-operative period. The main pathogens associated with SSIs were Gram-negative bacilli, methicillin-resistant *S. aureus* (MRSA), and methicillin-susceptible *S. aureus* (MSSA) (Durand et al., 2015). Notably, SSIs caused by MRSA are known as serious complications occurring in the post-operative period associated with increased hospital stay (Lin et al., 2017). Therefore, some decolonization protocols focused on MSRA, which are worth considering clinically (Kavanagh et al., 2014; Veve et al., 2017). The study conducted by Yang et al. demonstrated that in patients undergoing clean-contaminated surgery with free flap reconstruction, opportunistic pathogens, such as *P. aeruginosa* and *Enterococcus faecalis*, are more frequently responsible for SSIs than typical oral commensals (Yang et al., 2013). One of the methods used to treat SSIs may be the administration of tetracycline for 48 h after surgery due to oral cancer (Funahara et al., 2017). Some trials try to apply post-operative synbiotics in patients with HNCs to prevent surgical complications. Unfortunately, no significant improvement was revealed (Lages et al., 2018). Surgery itself might change the oral microbiome. It was shown that the salivary microbiome profile is abundant in bacteria as *Streptococcus anginosus*, *Abiotrophia defectiva*, and *Fusobacterium nucleatum* in patients with OSCC compared to that of the healthy controls. Surgical intervention induced a significant decrease in alpha diversity and an increase in the variability of the microbiome. Moreover, this change was still noticeable even after 2 years (Mäkinen et al., 2023). One the other hand, opposite conclusions were reached in the study by Schuurhuis et al., which showed the lack of change in microbiota composition associated with surgery, whereas radiotherapy with or without chemotherapy caused an increase in the number of opportunistic pathogens (Schuurhuis et al., 2016). Another study indicated that post-surgical restoration of some bacteria may improve the outcomes. It proved that post-surgery restoration of *Prevotella* 7 has a positive influence on survival. Specifically, the decreased relative abundance of *Capnopcytophaga*, *Prevotella* 7, and *Leptotrichia* as well as the increase in relative abundance of *Streptococcus* and *Rothia* were associated with a better 3-year disease-specific survival (Chan et al., 2021). Surgical resection of the tongue changes the salivary microbiome, which has been shown in the study of Kageyama et al. (2020). This study included 25 patients with tongue cancers. Sample swabs were collected from stimulated saliva in the pre- and post-operative period. The increasing amount of bacterial species from dental plaque regarding also periodontal pathogens was observed after surgical treatment (Kageyama et al., 2020).

To sum up the surgery aspects, surgical interventions are pivotal in the management of HNCs. It is important to prepare patients for surgery regarding also microbiome aspects. The maintenance of an appropriate nutritional status and modulation of microbiome through the administration of probiotics may significantly affect the final outcome of surgery (Nogal et al., 2022).

## 4 Immunotherapy

HNSCC is considered a disease with immunosuppressive character, and pembrolizumab and nivolumab have been registered in the recurrent or metastatic setting. Pembrolizumab is also approved as the first line of treatment. However, the majority of patients do not benefit from the treatment (Gavrielatou et al., 2020; Obradovic et al., 2022). Therefore, there is a need to investigate all possible mechanisms of resistance to mitigate the impact on tumor response. Moreover, there is also a need to find the predictive signature of response to immunotherapy. The microbiome seems to be both a promising target of treatment and a biomarker. Many studies have shown that changes in the microbiome composition can lead to cancer and affect the response to treatment (Roy and Trinchieri, 2017; Sobocki et al., 2021; Fasano et al., 2022; Sobocki et al., 2022), including chemotherapy and immunotherapy (Irfan et al., 2020; Zhou et al., 2021; Shiravand et al., 2022). Moreover, the microbiome composition is affected by alcohol consumption, which is a risk factor for HNCs (Fan et al., 2018; Fasano et al., 2022). However, the connection between microbiome and HNCs has not been investigated in a comprehensive way, and the literature is limited. The biggest clinically oriented study investigating the predictive role of microbiome in response to PD-L1 inhibitors is CheckMate141, which analyzed the saliva samples and oral microbiota. However, no significant correlation was observed (Ferris et al., 2017). On the other hand, some preclinical studies still show the potential of the microbiome (Gutiérrez Calderón et al., 2021). The study by Hu et al. indicated that the presence of *Luteibacter*, *Flammeovirgo*, and *Lachnoclostridium* was correlated with total T-cell receptor reads, number of clones, leukocytes, and CD8+ T-cell infiltration suggesting their potential role in tumor microenvironment and immunotherapy response regulation (Hu et al., 2023). Another retrospective study by Preissner et al. conducted in a group of 3,651 patients showed that administration of antibiotics decreased the immunotherapy effectiveness suggesting the major mechanism in gut microbiota changes (Preissner et al., 2023). Hu et al. proposed the mechanism in which microbes might increase chemokine levels in the tumor microenvironment, parallelly attracting T cells and increasing T-cell infiltration, and mediating the response to immune checkpoint inhibitors (ICIs) in HNCs (Hu et al., 2023). Mann et al., in the in-cell line study, showed that microbiota via Toll-like receptor 2 may directly modulate the expression of PD-L1 in HNCs. In addition, the effect of the gut microbiome in response to ICIs was proven in many different types of epithelial tumors (Routy et al., 2018; Zheng et al., 2019; Hamada et al., 2023). Currently, some studies registered in the Clinicaltrials.gov registry try to address directly or indirectly the issue of the link between microbiome in immunotherapy response (e.g., NCT05375266: recruiting, NCT05083416: active, not recruiting), which ought to be followed. The future should bring the answer to the question on whether the microbiome plays a significant role in immunotherapy response and is a promising and valuable target for treatment.

## 5 Chemotherapy

As was mentioned above, oral mucositis is associated with dysbiotic changes in the oral cavity. Oral mucositis can be induced not only through radiotherapy but also through chemotherapy (or combined chemoradiotherapy). In the study of Hong et al., it was shown that oral mucositis is associated with the exposure to 5-fluorouracil-based chemotherapy (Hong et al., 2019). Additionally, the alterations in the oral microbiome were detected through the increasing numbers of *Prevotella oris* and *F. nucleatum* with pathogenic properties. The modulation of the oral microbiome through the administration of probiotics and an appropriate dental products seems to be promising. It should be emphasized that the interaction between chemotherapy and microbiome in the case of HNCs has not been studied. Most of the studies describing that context are focused on oral mucositis, which is a cytotoxic effect of chemotherapy. It could be interesting to analyze the role of the oral microbiome as a community or population of microbes/particular microbe in response to chemotherapy. For instance, recently, it was detected that *F. nucleatum* is able to promote chemoresistance in the case of esophageal squamous cell carcinoma (Zhang et al., 2023). Some bacteria or fungi can be considered as biomarkers, which allow predicting the efficiency of chemotherapy.

## 6 Future directions

There are some directions for the future that should be taken into consideration during the next studies design as follows: (1) Most studies that discuss the link between the microbiome and HNCs are conducted with a small sample size. Therefore, there is a need to design multi-center clinical trials with a larger sample size, which would allow obtaining more conclusive and significant results. (2) Studies indicated the potency of probiotics in the reduction of oral side effects of anti-cancer management. Nevertheless, it is recommended to conduct double-blind, randomized, and placebo-controlled clinical trials. Moreover, the results of studies should clearly show which probiotic strain can be useful in this context due to the fact that the properties/activities of probiotics depend on the probiotic strain. (3) The regular examination of premalignant lesions is strongly recommended. Oncologists should cooperate with dentists to prepare, with the highest possible quality, the patients to the introduction of anti-cancer treatment. It means that every patient who is qualified to undergo anti-cancer treatment should undergo dental examination. It should be emphasized that dental care is extremely required also during and in the post-management period. (4) The oral microbiome may be used as a biomarker allowing the detection of cancer in the early stage. Nevertheless, it requires further studies regarding multiple factors that can affect the potency.

## 7 Conclusions

The group of patients with HNCs are at increased risk of anti-cancer treatment-associated complications especially in the case of high-dose radiotherapy. It seems that the dosage of radiation on the oral mucosa is crucial (and more important than initial tumor location) in the development of oral mucositis strongly affecting the nutrition and quality of life of the patients. The identification of oral microbiota changes may allow to modulate it in a eubiotic signature and consequently to relieve that symptom. Nevertheless, the complications that can occur in the oral cavity are more complicated, such as osteoradionecrosis, and may be developed in a long period after treatment. Therefore, there is a strong need to introduce an appropriate dental care prior, during, and in the post-management period. It can allow reducing the incidence and significance of treatment-associated oral complications. Additionally, the complex treatment regarding also the administration of probiotics seems to be promising in these patients. The insights into anti-cancer management and oral/intestinal microbiome in HNCs may provide modern and fresh approaches for clinicians.

## Author contributions

JM: Conceptualization, Writing – original draft. KK-S: Conceptualization, Writing – original draft. BS: Writing – original draft. ID: Writing – original draft. LK: Conceptualization, Supervision, Writing – review & editing. ES: Supervision, Writing – review & editing.
